# Effects of an inhibitor of the SHH signaling pathway on endometrial cells of patients with endometriosis

**DOI:** 10.1186/s12860-022-00426-5

**Published:** 2022-08-06

**Authors:** Yanan He, J. Wang, Xinyan Jiang, Jianhua Gao, Yan Cheng, Tian Liang, Jun Zhou, Liyuan Sun, Guangmei Zhang

**Affiliations:** Department of Gynaecology, The First Affiliated Hospital of Harbin Medical University, Harbin, China

**Keywords:** Endometriosis, Sonic hedgehog, Signaling pathway

## Abstract

**Background:**

Endometriosis is one of the most common gynecological diseases, and seriously reduces the quality of life of patients. However, the pathogenesis of this disease is unclear. Therefore, more studies are needed to elucidate its pathogenesis. Our previous publication found that the Sonic Hedgehog (SHH) signaling pathway was activated in endometriosis. This study tested whether SHH signaling in endometrial stromal cells (ESCs) was critical for the pathogenesis of endometriosis.

**Methods:**

To examine the effect of inhibiting the SHH signaling pathway on endometriosis, we first isolated ESCs from eutopic endometrial tissues of patients with or without endometriosis and identified the extracted cells by morphological observation and immunofluorescence. Then, we treated ESCs with the GLI inhibitor GANT61 and used CCK-8, wound healing and invasion assays to detect cell activities, such as proliferation, invasion and metastasis. Furthermore, we detected the expression of key proteins and proliferation markers of the SHH signaling pathway in the lesions of nude mice using immunochemistry.

**Results:**

We demonstrated that higher concentrations of GANT61 decreased the proliferation rate and migration distance of ESCs. We observed that GANT61 inhibited the invasion of ESCs. In addition, blockage of the SHH signaling pathway significantly reduced cell proliferation in vitro.

**Conclusions:**

Our study suggested that inhibition of the SHH pathway is involved in cell proliferation and invasive growth in the pathogenesis of endometriosis.

## Introduction

Endometriosis is a common gynecologic disease with a high risk of recurrence, that is characterized by the existence of endometrial tissues outside of the uterus [1, 2]. The abnormal growth of endometrial tissues can be found in the peritoneal cavity, cervix and fallopian tubes, leading to pelvic pain, dysmenorrhea and infertility [3, 4]. Although endometriosis seriously affects the quality of life of patients, the mechanism of the pathogenesis of endometriosis is largely unclear. To date, studies have focused on retrograde menstruation, estrogen-dependent glycoproteins, cytokines, adhesion molecules and angiogenic and growth factors, which are all associated with the pathogenesis of endometriosis and the development of endometriotic lesions. Retrograde menstruation was proposed by Sampson but has been called into question and challenged [5, 6], thus, other causative factors likely play roles in the occurrence and development of this disease.

Sonic Hedgehog (SHH), a mammalian member of the Hedgehog family (SHH, Indian Hedgehog and Desert Hedgehog) shares a common signaling pathway [7, 8]. HH ligand binding to the Patched (Ptc) transmembrane protein, activates the SHH pathway by relieving Patched1-dependent inhibition of Smoothened (SMO) signal transduction [9]. Subsequently, suppressor of fused (SUFU) and the GLI zinc finger family members (GLI2, GLI3) transmit the activated signal into the nucleus to regulate the expression of target genes, such as GLI1 and vascular endothelial growth factor. Accumulating evidence has shown that aberrant activation of the SHH signaling pathway plays a protumorigenic role in various types of gynecological cancers by enhancing cell proliferation, stem cell maintenance, cell differentiation and angiogenesis [10, 11]. Similar to cancers, endometriosis also develops through the dysregulation of cellular pathways regulating sensitivity to growth stimulation, cell proliferation and replication, angiogenesis and tissue invasion and metastasis. However, the role of the SHH signaling pathway in endometriosis still needs to be elucidated. Matsumoto et al. confirmed that recombinant SHH protein could enhance the proliferation of mouse endometrial mesenchyme cells in vitro [12]. Our previous research indicated that the SHH pathway was upregulated in eutopic endometrial tissues of endometriosis. Furthermore, comparison of revised AFS scores showed that SHH, SMO, GLI1 and GLI3 had significantly increased expression levels in patients with advanced disease (III-IV) [13].

In this study, we used the GLI inhibitor GANT61 to inhibit the SHH pathway and explored the effect of this pathway on the pathogenesis of endometriosis. Our purpose was to demonstrate the role of the SHH pathway in the proliferation, migration and invasion of endometrial stromal cells (ESCs) from eutopic endometrium derived from patients with endometriosis. Additionally, identifying new pathway mechanisms and providing new targets for clinical treatment are needed. Nevertheless, little is known about whether activation of the SHH pathway promotes the occurrence and development of endometriosis. Moreover, the SHH signaling pathway has also been suggested to regulate the cell cycle [14, 15], therefore, further elucidation of the underlying mechanisms of the SHH pathway in the biological function of ESCs is needed.

## Participants, materials and methods

### Participants

Human endometrial tissues were obtained from ten women aged 29–36 (32.3 ± 2.7) years undergoing surgery for idiopathic infertility in the First Affiliated Hospital of Harbin Medical University (Harbin, PR China). Eutopic endometrial tissues were obtained from ten patients aged 26–42 (34.3 ± 7.6) years undergoing surgery for endometriosis. None of the patients had received any hormonal therapy prior to surgery within six months. Women suffering from cancers, benign ovarian cysts other than endometriomas, perioperative pelvic inflammatory disease, or endometrial polyps were excluded from this study. All patients signed an informed consent form prior to recruitment and the study protocol was approved by the Ethics Committee of Harbin Medical University (202106). All experimental methods were carried out in accordance with the approved guidelines of Harbin Medical University. All patients gave their written informed consent prior to study inclusion.

### Animal experiments

All animal experiments were conducted using female Balb/c nude mice aged approximately 5 weeks and weighing 17–19 g. These mice were purchased from Charles River Laboratories in Beijing (No. 11400700316857), and the animal experiments were performed in strict accordance with the guidelines for the Care and Use of Laboratory Animals of the Harbin Medical University Ethics Committee. All procedures were approved by the Committee on the Ethics of Animal Experiments of Harbin Medical University. All efforts were made to minimize animal suffering. All cell protocols were approved by the Harbin Medical University Ethics Committee.

### Isolation and culture of ESCs

Endometrial tissues were collected and washed with ice-cold medium (DMEM/F-12 1:1) (Hyclone, USA) containing 10% fetal bovine serum (Ausbian, USA) and 1% penicillin-streptomycin (Gibco, USA). The samples were transported to the laboratory on ice within 2 h. The endometrial tissues were cut into smaller pieces and digested in type IV collagenase (Life Technologies, Carlsbad, CA, USA) at 37 °C for 60–90 minutes. The cell suspension was passed once through a 70-μM sieve (HEAD, Beijing, China) to remove debris and glandular epithelial cells. The filtrates were then centrifuged at 800 rpm for 5 minutes at room temperature. The isolated cells were maintained in the medium mentioned above at 37 °C and 5% CO_2_. The medium was replaced after 2–3 days to remove nonadherent cells. Cells were subcultured on new plates at a 1:2 ratio and marked as passage 1 (P1) [16, 17]. Cells from P3-P5 were used for the experiments.

### Observation of ESC morphology

The morphology of ESCs from different passages was observed by inverted light microscopy (Olympus, Japan).

### Immunofluorescence

Serum-starved ESCs were seeded on cover glass slides, fixed with 4% paraform aldehyde for 15 min, and permeabilized with 0.1% Triton X-100. The purity of ESCs was detected by separately immunostaining for the epithelial marker cytokeratin 7 (CK 7) (33,060 M, Biosis, China) and stromal marker vimentin (VIM) (0756R, Biosis, China). We used 4′,6′-diamidino-2-phenylindole (DAPI) immunofluorescence to identify ESC nuclei. Cells were incubated with fluorescein isothiocyanate (FITC)-phalloidin (for F-actin staining, Sigma, USA) at room temperature for 40 minutes before incubation with the primary antibodies anti-CK 7 and anti-VIM overnight at 4 °C.Cell nuclei were stained with DAPI (Thermo Fisher Scientific). Immunofluorescence signals were observed using an inverted light microscopy (Olympus, Japan) [18]. The areas with CK 7 and VIM were computed using ImageJ software. At least 100 cells were analyzed from triplicate cover slides in each sample, and experiments were repeated with samples from five different individuals.

### Cell proliferation assay

Cell proliferation was assessed by the Cell Counting Kit-8 (CCK-8; Dojindo, Japan) assays. To determine whether the SHH signaling pathway effected ESC proliferation, we applied the SHH signaling pathway inhibitor GANT61. After 48 hours, cells were seeded in 96-well plates (4000 cells per well) stimulated with different concentrations of GANT61 (SIGMA, USA) in 100 μL of full culture medium. The cells were tested in the absence (NC) and presence of 10 μmol/L, 20 μmol/L and 30 μmol/L inhibitor of GANT61. Ten microliters of CCK-8 solution was added to each well. Absorbance was read at a wavelength of 450 nm by a microplate reader (ELX800; Bio-Tek, Ameria). The proliferation rate was derived from the cell index, which was calculated as the difference between the well with only cells minus the well with only culture media, divided by the nominal value. Three independent experiments were performed in triplicate.

### Wound healing assay

To examine the migratory capacity of ESCs, we conducted a scratch wound assay. Cells cultured with GANT61 (10 μmol/L, 20 μmol/L and 30 μmol/L) were seeded in six-well culture plates with serum-containing medium and cultured until the cell density reached 90–95% confluence. An artificial homogeneous wound was created by scratching the monolayer with a sterile 200 μL pipette tip. After scratching, the cells were washed with PBS and then cultured with serum-free DMEM F12 1:1 media for 48 hours. Images of cells migrating into the wound were captured at 0 and 48 hours using a microscope (EVOS, USA). The assay was performed in triplicate [19]. Three independent experiments were performed in triplicate.

### Transwell invasion assay

Transwell assays were used to assess cell invasive capacity. Cell invasion assays were carried out using a BioCoat Matrigel Transwell chamber (BD, Franklin Lakes, NJ, USA) with a pore size of 8.0 μm. The inserts were placed in 24-well plates containing 700 μL of DMEM F12 1:1 medium for 30 minutes in a humidified 37 °C incubator under 5% CO_2_ before seeding the cells. GANT61 (30 umol/L) was used to block the SHH signaling pathway, and after 48 hours, 5 × 10^4^ cells in each group resuspended in DMEM F12 1:1 medium containing 5% FBS were placed in each chamber. The lower compartment was loaded with full media containing 15% FBS as the nutritional attractant. After incubated at 37 °C for 48 hours, noninvaded cells were scraped off with a cotton swab. The translocated cells on the bottom of the upper chamber membrane were fixed with 5% formaldehyde and stained with 1% Giemsa stain. The number of cells that penetrated the upper compartment of the Transwell chamber was determined under an inverted microscope. Five fields of fixed cells were randomly chosen and counted under a light microscope [19].

### Immunohistochemistry

All tissues were fixed in 10% formaldehyde, embedded in paraffin and cut into 4 mm sections. Immunohistological staining was conducted by boiling the sections in 10 mM citric acid, pH 7.0. The slides were incubated with a polyclonal rabbit antibody (1:200 dilution; Biosis) for 2 hours at 37 °C. The sections were washed in phosphate-buffered saline (PBS) three times and then incubated with mouse anti-rabbit secondary antibody for 40 minutes at 37 °C Peroxidase substrate containing 3,3′-diaminobenzidine tetrahydrochloride chromogen was added to the sections for 2 minutes to develop the reaction. All slides were examined and scored by two independent pathologists who were blinded to both the clinical and pathological data. The quantification of the selected proteins was performed using Image-Pro Plus 6.0 (Media Cybernetics). Scoring was carried out for the mean density (ratio of integrated optical density SUM/area) [19].

### ESCs and eutopic endometrium tumourigenicity analysis

Mice were kept on a 12 h light/dark cycle and provided sterile food and water. The mice were allowed to acclimate to specific pathogen-free (SPF) conditions before experiments. The mice were randomly separated into 2 tumorigenicity groups (*n* = 5 per group), the ESC and eutopic endometrium groups. For further study of the function of the SHH signaling pathway in vivo, a mouse model of experimental endometriosis was established by injecting NS with 0.5 cm^3^ in size of eutopic endometrial fragments into the right subcutaneous scapular tissue and injecting the contralateral side with 0.2 ml of normal saline (NS) as the negative control. Lesions were monitored daily in both groups. After 40 days, the mice were sacrificed by cervical dislocation. Both the left and right subcutaneous scapular tissues were collected. Macroscopic observation and H&E staining were used to assess lesion formation [20].

### GANT61 treatment of ESCs in the endometriosis model in vivo

Human eutopic endometrial tissues were obtained from patients with endometriosis as described above. Mice were maintained on a 12 h light/dark cycle and were provided with sterile food and water. The mice were allowed to acclimate to SPF conditions before experiments. Twenty mice received a single subcutaneous injection of a 0.5 cm^3^ eutopic endometrial fragment in 0.2 ml of NS into their back. Seven days later, when the endometriosis model was confirmed, the mice were randomly divided into two groups (*n* = 10 per group), the GANT61 group and the control group. GANT61 was subcutaneously injected into experimental group mice, while NS was injected into the controls. In the GANT61 group, 30 umol GANT61 in 0.3 ml of NS was administered intravenously into the tail vein. The mice in the control group were only injected with 0.3 ml of NS. The injections were performed weekly. The animals were sacrificed one week after the third injection, and the endometriotic lesions of the two groups were collected to detect the effect of GANT61 on lesion reduction [20].

### Statistical analysis

All statistical analyses were performed using SPSS 19.0 (SPSS, Inc., Chicago, IL). Continuous variables are expressed as the mean ± standard deviation. Differences between groups were evaluated using the independent samples Student’s t test. Completely random design analysis of variance was performed to test the significance of the migration of ESCs in response to different GANT61 doses. Data from the invasion assay were assessed by paired T tests. Differences were considered statistically significant at *P* < 0.05.

## Results

### Isolation, culture and immunofluorescence identification of ESCs

**Fig. 1:**
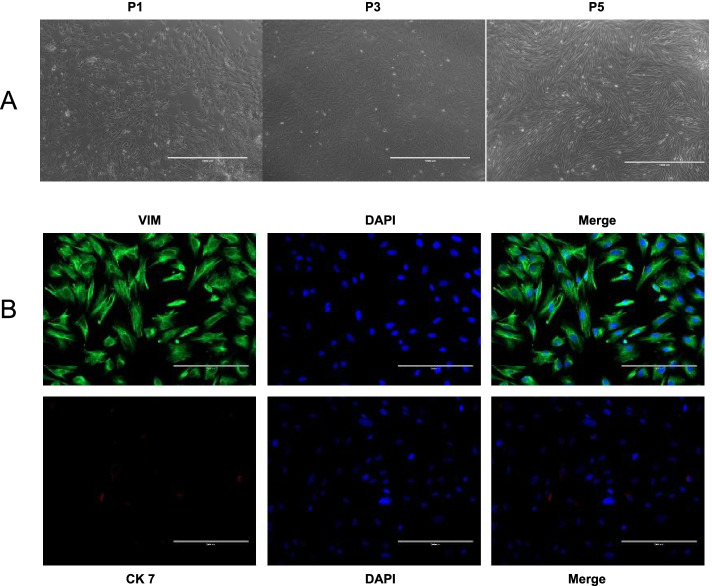
Isolation, culture and morphological observation of ESCs. **A** Isolation, culture and morphological observation of ESCs. Scale = 1000 μm. **B** Immunofluorescence identification of the type and purity of cultured cells. Scale = 200 μm

ESCs were successfully extracted from endometral tissues by the centrifugal adherent method. After 5 to 7 days in primary culture, adherent cells began to form cell clones. Primary ESCs exhibited a short polygonal or fusiform morphology, which gradually became a fibroblast-like spindle shape with an increasing number of passages. Cells from passages 3–5 (P3-P5) showed a relatively homogenous morphology, long spindle shape and a swirling arrangement (Fig. 1A).

Cellular immunofluorescence detection was used to identify the type and purity of cultured cells. CK 7 was mainly localized in the endometrial glandular epithelial cells, and VIM was in the endometriotic stromal cells. As shown in the Fig. 1B, endometrial glandular epithelial cell cytoplasm was positive for CK 7, while endometrial glandular epithelial cell nuclei were positive for VIM. We observed a large amount of VIM-positive staining and a small amount of CK 7-positive staining by inverted fluorescence microscopy. The experiments that measured VIM-positive cells at more than 95% provided clear evidence that ESCs were successfully isolated.

### Inhibition of the SHH signaling pathway reduces the proliferation of ESCs

**Fig. 2:**
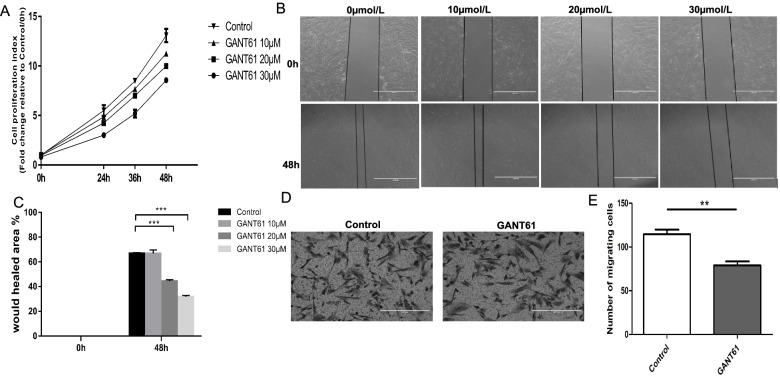
Inhibition of the SHH signaling pathway reduces the ability of proliferation, migration and invasion of ESCs. **A** Effects of different concentrations of GANT61 (10 μmol/L, 20 μmol/L and 30 μmol/L) on the proliferation of ESCs. **B** Effects of different concentrations of GANT61 on the migration of ESCs. Scale = 1000 μm. **C** Effect of GANT61 (30 mol/L) on the invasive ability of ESCs. **D** Quantitative plots of the number of invasive cells in the control group and the GANT61 group

The ESCs stimulated with different concentrations of GANT61 showed a decreased proliferation index compared with the controls (Fig. 2A). A significant difference was observed when GANT61 was added at a concentration of 30 μmol/L. These results were statistically significant. The CCK-8 assay demonstrated that stimulation with GANT61 diminished the proliferation of ESCs, especially at a concentration of 30 μmol/L.

### Reducing the migratory and invasive capability of the ESCs by inhibiting the SHH signaling pathway

Would healing assays showed that the inhibitor of the SHH signaling pathway reduced the migration of ESCs (*P* < 0.01) compared to the controls (Fig. 2B). As shown in Fig. 2C, 20 μM and 30 μM GANT61 resulted in a smaller scratch area after 48 h than that of the controls. The average values of the scratched areas with GANT61 at 20 μM and 30 μM were 44.457 and 21.77, respectively. In addition, Transwell assay showed that the invasion rate was significantly reduced by the inhibitor GANT61 (Fig. 2D). The number of invading cells was statistically significantly higher in the control group than in the experimental group. As shown in Fig. 2E, a difference in the invasion rate was observed before and after application of GANT61. Similar to the results of the proliferation assay, these findings demonstrated that stimulation with GANT61 decreased the migratory and invasive capacity of ESCs.

### Construction and characterization of a nude mouse xenograft model

**Fig. 3:**
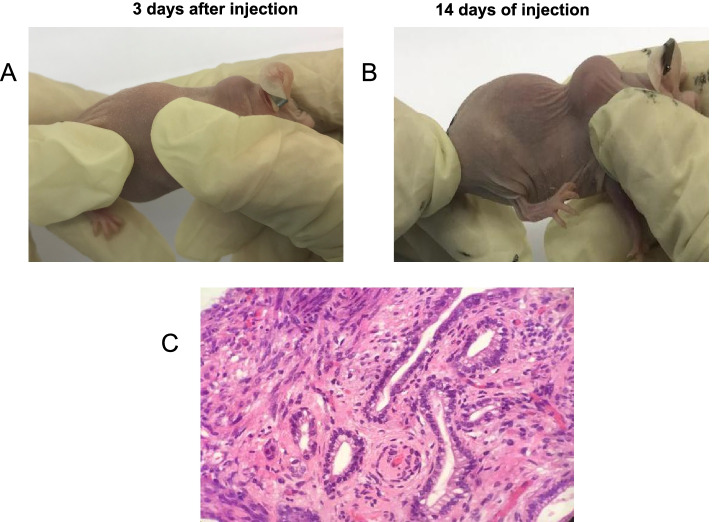
Subcutaneous xenografts in nude mice. **A** Macroscopic observation of subcutaneous xenograft tumor formation after 3 days. **B** Macroscopic observation of subcutaneous xenograft tumor formation after 14 days. **C** H&E of subcutaneous xenograft tumor formation in control nude mice and GANT61 nude mice. H&E staining (400 ×)

Two days after injection of eutopic endometrial fragments, nude mice developed a soft rash at the injection site. After 1 week, a slightly soft round mass was observed at the injection site of the right subcutaneous scapular tissue in the nude mice, and the visible lesions increased in size over time. All mice were sacrificed 21 days post-injection by cervical dislocation and multiple lesions were removed for further analysis. By macroscopic observation (Fig. 3A), we confirmed the endometriotic lesions in the nude mice. Moreover, endometrial glands and stromal structures were observed in the HE-stained sections under a microscope (Fig. 3C). Through visual observation (Fig. 3A-2) and H&E staining (Fig. 3B-3), we found that the right side of the five nude mice had formed endometrioid tissue, so the nude mouse xenograft model was successfully constructed.

### Detecting key SHH signaling pathway proteins and Ki67 in ectopic lesions in the GANT61 group and the control group

**Fig. 4:**
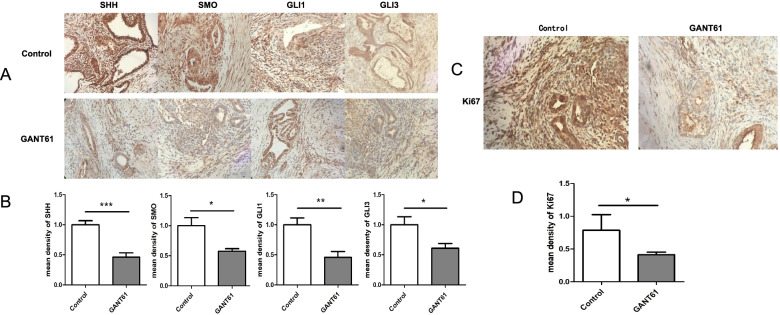
Expression of SHH, SMO and GLI1 and GLI3 in ESCs. **A** The protein expression of SHH, SMO, GLI1 and GLI3 in subcutaneous xenograft tumor lesions of nude mice in the control group and the GANT61 group. **B** Quantitative mean density of SHH, SMO, GLI1 and GLI3 in the control group and the GANT61 group. **C** The protein expression of Ki67 in subcutaneous xenograft tumor lesions of nude mice in the control group and the GANT61 group (400 ×). **D** Quantitative mean density of KI67 in the control group and the GANT61 group (400 ×)

The protein expression levels of the experimental group, including those of SHH, SMO, GLI1 and GLI3, were significantly lower than those of the controls (Fig. 4A and B). Ki67 is a nuclear antigen expressed in the mid-G1, S, G2 and M phases of the cell cycle, and it serves as a marker of cell proliferation. Ki67 is closely associated with mitotic cellular chromosomes and centrally involved in cell proliferation [21, 22]. Through a series of in vitro experiments, we found that blocking the SHH signaling pathway decrease the proliferation of ESCs (Fig. 4C and D). The in vivo proliferation experiments were applied to further validate the effect of blocking SHH signaling on Ki67 protein expression. In addition, using an in vitro assay, we found that blockade of the SHH signaling pathway can significantly reduce cell proliferation and that the SHH pathway is essential for cell proliferation in endometriosis.

## Discussion

Endometriosis has similar characteristics to malignancies, including excessive cell proliferation, invasion, metastasis and recurrence. The SHH signaling pathway has a diverse range of biological functions, including the promotion of cell proliferation and differentiation along with the induction of angiogenesis and cell migration [23]. Understanding the molecular mechanism by which the biological behaviors of ESCs are regulated in endometriosis would improve our understanding of the etiology and pathogenesis of endometriosis.

Similar to other pathways, the SHH signaling pathway was significantly related to changes in hormone levels in females. Monsivais et al. [24] showed that the occurrence of endometriosis was related to the steroid signaling pathway, especially the estrogen and progesterone signaling pathways. Zhang et al. [25] found that this elevated expression of SHH signatures was associated with progesterone receptor positivity in human trophoblasts. This result suggested that the SHH signaling pathway may play an important role in endometriosis. In our previous study, we investigated the effect of the SHH signaling pathway on the development of endometriosis. The eutopic endometrium was compared with the normal endometrium using qRT-PCR and immunohistochemical staining. SHH, SMO, GLI1 and GLI3 expression was strongly increased with clinical stages in the eutopic endometrium, which suggested that the SHH signaling pathway was abnormally activated in endometriosis [13]. Additionally, the SHH signaling pathway might have important implications for the development and prognosis of tumor diseases. Noman et al. [26] confirmed that elevated levels of the SHH signaling pathway were observed in breast patients who had a significantly higher risk of recurrence and metastasis and had worse survival than patients with progressive metastatic breast cancer. Gomes et al. [27] found that the group with craniopharyngiomas had more rapid cancer progression and poorer five-year survival outcomes compared to the control group. Therefore, we further investigated the mechanism of the SHH pathway in endometriosis.

According to our results, immunohistochemistry showed higher expression levels of nuclear GLI1 and GLI3 not only in endometrial stromal cells but also in endometrial glandular epithelial cells. Expression in endometrial glandular epithelial cells is a marker of stem or progenitor cells. Based on both the importance of the SHH signaling pathway in various cancers and the tumoroid characteristic of eutopic endometrial cells, we speculated that differential expression of key SHH signaling pathway proteins may change the biological behavior of eutopic endometrial cells.

Inhibitors targeting the SHH signaling pathway could decrease cell proliferation, migration, and invasion. Small molecule modulators of the SHH signaling pathway have been extensively investigated. Few studies have examined the terminal transcription factor GLI as a SHH pathway inhibitor to intervene in disease occurrence and development. Consequently, we assessed regulation of the SHH signaling pathway with the development of endometriosis. We examined the influence of the GLI transcription factor inhibitor GANT61 on endometriosis. GANT61 is an available and potent inhibitor of the terminal transcription factor GLI that not only induced the proliferation of tumor cells in vitro but also promoted cell apoptosis [28, 29]. In vitro, we demonstrated that the small molecule inhibitor GANT61 exerted inhibitory effects on the expression of GLI downstream target genes and blocked pathway transmission. We also showed that GANT61 inhibited the proliferation of ESCs by blocking the SHH signaling pathway. GANT61 could suppress ESC proliferation compared to that of the control group without treatment. Heard et al. demonstrated that KLF9 deletion increased proliferation and reduced apoptosis in a mouse model of endometriosis through the Hedgehog and Notch signaling pathways; however, there were no further studies on Hedgehog signaling in endometriosis [30]. Additionally, we explored this problem further through in vivo experiments. The protein expression levels of the experimental group after GANT61 injection, including those of SHH, SMO, GLI1 and GLI3, were significantly lower than those in the control group. Barricading the SHH signaling pathway, the expression of the cell proliferation-related protein Ki67 in the experimental group showed a stronger decrease than that in the control group in the animal model of endometriosis. In vitro, the cell proliferation rate of ESCs decreased as the dose of GANT61 increased. The studies above demonstrated that the SHH signaling pathway was activated, and the proliferation of endometrial stromal cells was weakened if the pathway was blocked. Thus, we believe that blockade of the targeted factor of the SHH signaling pathway will decrease the expression and activation of the pathway and will also inhibit the development of endometriosis.

Cell migration is required for various physiological and pathological processes, such as wound repair, angiogenesis, inflammatory responses, immune cell phagocytosis and invasion and metastasis of cancer cells [31, 32]. The invasion and migration of endometrial cells play an important role during endometriosis development [33, 34]. We examined the effect of the SHH signaling pathway on the migration and invasion of ESCs. In our study, blockage of the SHH signaling pathway resulted in a shorter migration area of ESCs and a lower number of invading cells compared with the controls. A previous study showed that the antimigratory effect was associated with the inhibition of HH signaling by SMO inhibitors, such as NVP-LDE-225 and GDC-0449, both of which have been approved for clinical use [35, 36]. However, these drugs might be ineffective against nonclassical GLI activation pathways. In addition to SMO inhibitors, direct inhibitors of GLI and/or inhibitors of signaling pathways involved in the noncanonical HH-GLI pathway may be required for effective treatment. Additionally, Souzaki et al. showed that the SHH signaling pathway advanced the progression from ductal carcinoma in situ (DCIS) to invasive ductal carcinoma (IDC) [37]. Su et al. [38] suggested that the HH signaling pathway enhanced the viability and invasion of gastric cancer cells.

The present research further extended the mechanistic study of the SHH signaling pathway in endometriosis. The results above provided a basis for an in depth understanding of the pathophysiology and a search for new therapeutic targets. Therefore, the SHH signaling pathway may serve as a novel target for endometriosis therapy in the future. These findings have significant implications for identification of nonhormone medications for the treatment of endometriosis. This study had some limitations. Due to the poor passaging ability of endometrial glandular epithelial cells, the Ishikawa cell line was generally used in the experiment, so endometrial glandular epithelial cells were not further studied in this report. Additionally, the number of experimental nude mice was relatively small. In the future, it will be necessary to expand the sample size of experimental nude mice to further study the clinical application value of GANT61 in the treatment of endometriosis.

Taken together, our previous research showed that the expression levels of SHH, SMO, GLI1 and GLI3 were higher in the control tissues than in the eutopic endometrial tissues [13]. Our study would provides a basis for a retrospective analysis of the endometrosis mechanism, which we expect to play a role in the treatment of endometriosis. The results of in vitro experiments showed that GANT61 had antiproliferative, anti-invasive and antimetastatic effects in ESCs by inhibiting the SHH signaling pathway. This study increased the understanding of the pathogenesis of endometriosis, and provided a molecular basis for further expanding experimental samples to conduct in vivo experiments to study the therapeutic effect of GANT61. Overall, this study provides new evidence for understanding the etiology of endometriosis and seeking novel clinical treatments.

## Data Availability

All data for this study are included in this article.
